# Molecular Mechanisms Underlying Increase in Lysine Content of Waxy Maize through the Introgression of the *opaque2* Allele

**DOI:** 10.3390/ijms20030684

**Published:** 2019-02-05

**Authors:** Wei Wang, Suzhen Niu, Yi Dai, Xinmi Zhai, Mingchun Wang, Yanqin Ding, Wenpeng Yang, Degang Zhao

**Affiliations:** 1 The Key Laboratory of Plant Resources Conservation and Germplasm Innovation in Mountainous Region (Ministry of Education), Institute of Agro-Bioengineering and College of Life Sciences, Guizhou University, Guiyang 550025, China; wwmaize@126.com (W.W.); niusuzhen@163.com (S.N.); daiyi96@126.com (Y.D.); www44pipi@163.com (X.Z.); dyqcyl@163.com (Y.D.); 2 The State Key Laboratory Breeding Base of Green Pesticide and Agricultural Bioengineering, Guizhou University, Guiyang 550025, China; 3 Guizhou Institute of Upland Food Crops, Guizhou Academy of Agricultural Sciences, Guiyang 550006, China; wangmingchun64@163.com

**Keywords:** *opaque2*, *waxy*, Transcriptom, RNA-Seq, *Zea mays* L.

## Abstract

The *opaque2* (*o2*) mutation in maize is associated with high lysine content in endosperm and good nutritional value. To improve the nutritional quality of waxy maize, the *o2* allele was introgressed into the *wxwx* line using marker-assisted backcrossing selection technology. The lysine content of *o2o2wxwx* lines was higher than that of the *wxwx* line. To reveal the mechanism of increasing lysine content through introgression of the *o2* in waxy maize, the transcriptome on kernels (18th day after pollination) of the *o2o2wxwx* and parent lines was analyzed using RNA-sequencing (RNA-Seq). The RNA-Seq analysis revealed 49 differentially expressed genes (DEGs). Functional analysis showed that these DEGs were mostly related to the catalytic activity and metabolic processes. The *O2* gene regulated multiple metabolic pathways related to biological processes (BP) and molecular function (MP) during waxy maize endosperm development. In particular, in the *o2o2wxwx* lines, the two genes that encode the EF-1α and LHT1 were up-regulated, but the gene that encodes sulfur-rich proteins was down-regulated, raising the grain lysine content. These findings are of great importance for understanding the molecular mechanism underlying the lysine content increase due to *o2* allele introgression into waxy maize.

## 1. Introduction

Corn (*Zea mays* L.) is one of the main fodder and food crops. The quality and taste of corn can directly affect healthy development in humans, livestock, and poultry. Lysine is an essential amino acid for monogastric animals such as humans, livestock, and poultry, and the amylopectin content in starch is closely related to food quality. In corn, the *waxy* (*wx*) mutation can increase grain amylopectin content to improve the eating quality, while the *opaque2* (*o2*) mutation can increase the lysine content to improve the nutritional quality. The lysine content in normal corn (<0.3%) is lower than the nutritional quality required for human consumption and food processing (>0.5%; the index was recommended by Food and Agriculture Organization of the United Nations (FAO), and the World Health Organization (WHO)) [1]. In 1964, Mertz et al. found that the *o2* mutation could significantly increase the lysine content of corn endosperm [2]. The *Opaque2* (*O2*) gene is located on the short arm of chromosome 7, and encodes a leucine zipper family transcription factor. This factor contains a basic domain (ACGT) that activates the expression of 22 kDa α-zein and 15 kDa β-zein genes [3]. The domain can also directly or indirectly regulate other non-zein protein genes, such as *b-32* and *b-70* [4]. Meanwhile, waxy corn, which possesses a good eating quality, was first found in China [5,6,7]. Collins described the characteristics of waxy corn for the first time in 1909 [8]: The grain was dark, smooth, and waxy, and the endosperm was composed of 95–100% amylopectin. The *Waxy1* (*Wx*) gene is located on the long arm of chromosome 9, and encodes granule-bound starch synthase I (GBSS-I). Additionally, it determines the amylose synthesis in maize endosperm and pollen [9,10,11,12,13]. The mutated endosperm was caused by genetic drift and its lysine content was not high enough (0.24–0.34%) to meet the nutritional quality requirements for human consumption and food processing [14].

To improve the nutritional quality for waxy corn, Misra et al. integrated the *wx* and *o2* genes into four inbred lines–Oh43, W64A, B37, and C103–with lysine and free amino acids being higher in the double mutants (*o2wx*) compared to the corresponding single mutants [15]. Zhang et al. acquired 18 high-quality maize inbred lines containing *wx* and *o2* genes that had lysine contents ranging from 0.36–0.54%; these were 1.15–27.06% higher than the lysine contents of the original parent lines [16]. Yang et al. integrated *o16* and *wx* genes to obtain 13 corn families; the lysine contents of these corn families were at least 16% higher than those of their original parents [17]. The literature indicates that lysine content can be increased without affecting the waxy property, through combining high-lysine genes and waxy genes [18]. The introgression of the *o2* gene into normal corn with different genetic backgrounds caused different degrees of transcriptional modifications [19]. The *o2* gene altered the physiological, biochemical, and proteomic properties of corn to some extent [14]; with the introgression of *o2* not only reducing the accumulation of prolamin, but also altering the expression of endosperm proteins associated with amino acids, biosynthesis, starch, stress response, and signal transduction [14,20]. However, the underlying molecular mechanisms of the *o2* gene that result in an increase in the lysine content of corn remain unknown.

In this study, an *o2* gene was introgressed into waxy corn by backcrossing using the marker-assisted backcrossing selection (MABS) technology. After foreground and background selections, two *o2o2wxwx* double recessive inbred lines, QCL8004_1 and QCL8004_2, were acquired. To understand the regulatory transcriptional network of waxy corn after *o2* gene introgression, kernels at 18 days after pollination (18th DAP) were used for transcriptional sequencing analysis. The transcriptional differences between the double recessive mutants (*o2o2wxwx*) and the waxy parent (*wxwx*) were compared. These results provide a foundation for studying the underlying molecular mechanisms of the *o2* gene introgression-driven improvements in lysine and tryptophan content.

## 2. Results

### 2.1. The Kernel Morphological Characteristics and Lysine Content of Pyramided Progenies

**Figure 1:**
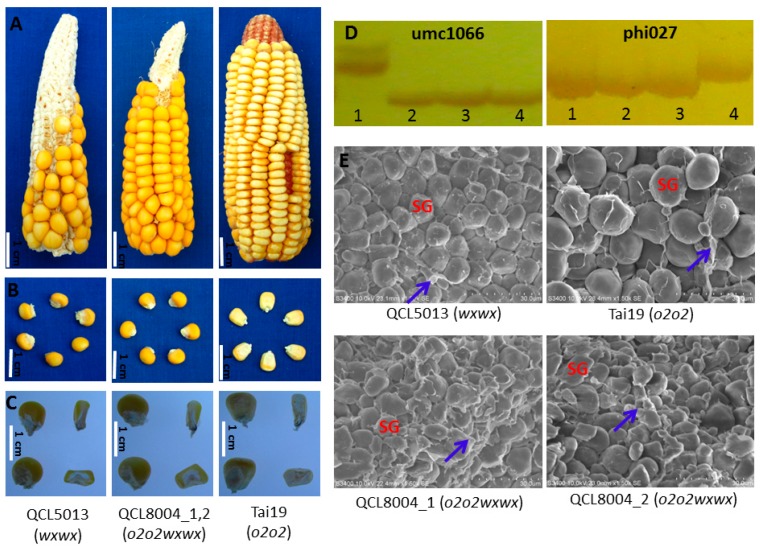
Phenotypic features of *o2o2wxwx* and recurrent parent. (**A**) Photographs of intact ears taken under normal light. (**B**) Photographs of mature kernels taken under normal light. (**C**) Light transmission of mature kernels on a light box, Bars = 1 cm. (**D**) Electrophoresis pattern of SSR marker for target gene *o2* and *wx*. Lane 1, QCL5013; Lane 2, QCL8004_1; Lane 3, QCL8004_2; Lane 4, Tai19. (**E**) Scanning electron micrograph for endosperms of QCL8004_1, QCL8004_2, QCL5013, and Tai19 at 1500× magnification, respectively, Bars = 30 µm. SG, starch granules; Blue arrows, matrix protein.

In this study, the grain phenotype and endosperm cross section of *o2o2wxwx* mutant lines and their recurrent parent were observed under natural light. The results showed that grain seeds of the recurrent parent and *o2o2wxwx* mutant were smooth, opaque, and waxy (Figure 1A,B). Meanwhile, there was a slight increase in the amount of farinaceous endosperm compared to the recurrent parent QCL5013 (Figure 1C). Two markers of simple sequence repeats (SSR) markers umc1066 and phi027 were used for *o2* and *wx* genes verifications, and were introgressed into QCL8004_1 and QCL8004_2 (Figure 1D). Under scanning electron microscopy (SEM), the starch granules of QCL8004_1 and QCL8004_2 had an irregular shape and arrangement, with a high density of matrix proteins that dispersed in the gap between starch granules, while those of QCL5013 and Tai19 were mostly elliptical and relatively smooth (Figure 1E). 

Lysine contents of QCL8004_1 and QCL8004_2, detected on mature kernels from the Inspection and Testing Center for Quality of Cereals and their Products, the Ministry of Agriculture and Rural Affairs of the People’s Republic of China, were 0.37% and 0.38%, respectively (Table 1). These values were higher than those of QCL5013 by 15.63% and 18.75%, respectively; meanwhile, their amylopectin contents of mature kernels were above 98%, which was equivalent to the recurrent parent (Table 1).

### 2.2. The Recovery Rate of the Genetic Background of o2o2wxwx Lines

**Figure 2:**
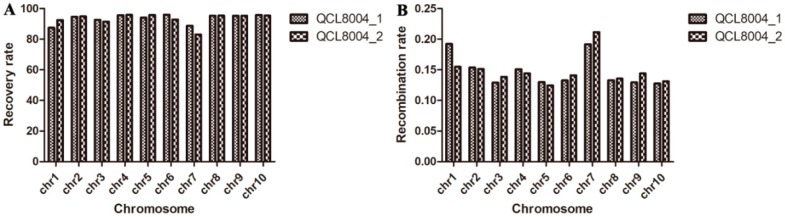
The background recovery rate (**A**) and recombination rate (**B**) of QCL8004_1 and QCL8004_2 in ten chromosomes.

In total, 55,229 single nucleotide polymorphism (SNP) markers were incorporated into the 55K SNP chip (Table S1), and 15,514 polymorphic SNPs were found between Tai19 (*o2o2*) and QCL5013 (*wxwx*). Based on the SNP markers, the genetic background recovery rates of QCL8004_1 and QCL8004_2 were 93.16% and 93.28%, respectively, which were higher than the theoretical rate (87.50%). Moreover, the background recovery rates of QCL8004_1 chromosomes based on the SNP markers ranged from 88.68–95.83% and the average rate was 93.54%. In comparison, the recovery rates of QCL8004_2 chromosomes ranged from 83.04–95.85% and the average rate was 93.23% (Figure 2A). These data indicate that QCL8004_1 and QCL8004_2 show higher background recovery rates than the theoretical rate (87.50%).

The chromosome recombination frequencies in the *o2o2wxwx* double recessive mutants were calculated by Graphical GenoTypes (GGT32). The results, shown in Figures S1 and S2, demonstrated that the recombination rates of different chromosomes differed slightly, and were relatively higher in chromosomes 1 (chr1) and 7 (chr7) (Figure 2B). Meanwhile, the recombinant fragment distributions in each chromosome of QCL8004_1 and QCL8004_2 differed slightly. Those fragments were relatively concentrated in chromosomes 1 (chr1), 3 (chr3), 4 (chr4), 6 (chr6), and 7 (chr7). In chr1, fragments were mainly concentrated in AX-86241431 (SNP, physical locations, 241.59 MB) to AX-90605656 (250.24 MB), and AX-91422403 (281.79 MB) to AX-90728414 (282.73 MB). In chr3, fragments were mainly concentrated in AX-91553625 (0.20 MB) to AX-91554402 (4.36 MB), and AX-91412879 (187.01 MB) to AX-90849007 (199.93 MB). In chr4, fragments were mainly concentrated in AX-91630097 (170.66 MB) to AX-86313874 (176.16 MB). In chr6, fragments were mainly concentrated in AX-86259944 (7.75 MB) to AX-90587704 (8.77 MB), and AX-90989242 (45.05 MB) to AX-91690814 (50.15 MB). In chr7, fragments were mainly concentrated in AX-91040704 (68.48 MB) to AX-123945296 (88.11 MB), and AX-86252028 (113.54 MB) to AX-91055672 (124.07 MB). This was related to the *o2* gene from the donor parent and the directional selection of high lysine.

### 2.3. The Quality of RNA-Seq and Biological Replicates

RNA-seq analysis showed that each sample generated an average of 22,962,789 clean reads (Table S2), the average mapping ratio to the reference gene was 81.77% (Table S3), and the average mapping ratio to the reference genome was 90.31% (Table S4). After quality control, the average Q20 (the percentage of the number of bases with a sequencing base mass value greater than 20 in the total number of bases in the original data) of all samples was 95.86% and the average Q30 (the percentage of the number of bases with a sequencing base mass value greater than 30 in the total number of bases in the original data) was 86.50% (Table S5), indicating good quality control and high utilization of sequencing data.

**Figure 3:**
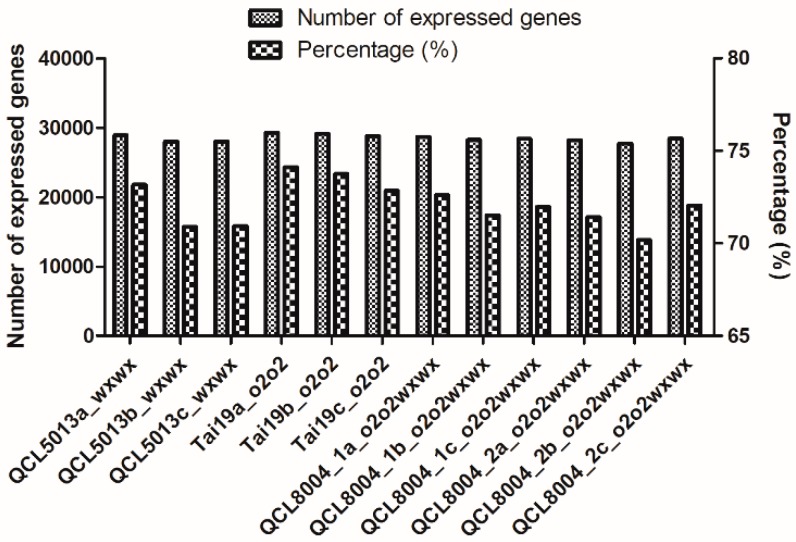
The number and percentage of identified genes in each sample.

Quantitative expression analysis of the sequenced genes was presented as fragments per kilobase million (FPKM; Table S6). Overall, 28307, 28451, 28128, and 29061 genes were detected in QCL5013, QCL8004_1, QCL8004_2, and Tai19, respectively, accounting for 71.67%, 72.03%, 71.21%, and 73.58% of the total genes of corn (Figure 3), indicating that most genes were covered.

Correlation among the three replicates of each sample was calculated according to the FPKM quantification result. The correlation coefficients of QCL5013, QCL8004_1, QCL8004_2, and Tai19 were 0.9778, 0.9897, 0.9746, and 0.9962 (Figure S3), respectively, indicating that the transcriptional sequencing data of the three replicates for each sample could be further analyzed.

### 2.4. Identification of Differentially Expressed Genes

**Figure 4:**
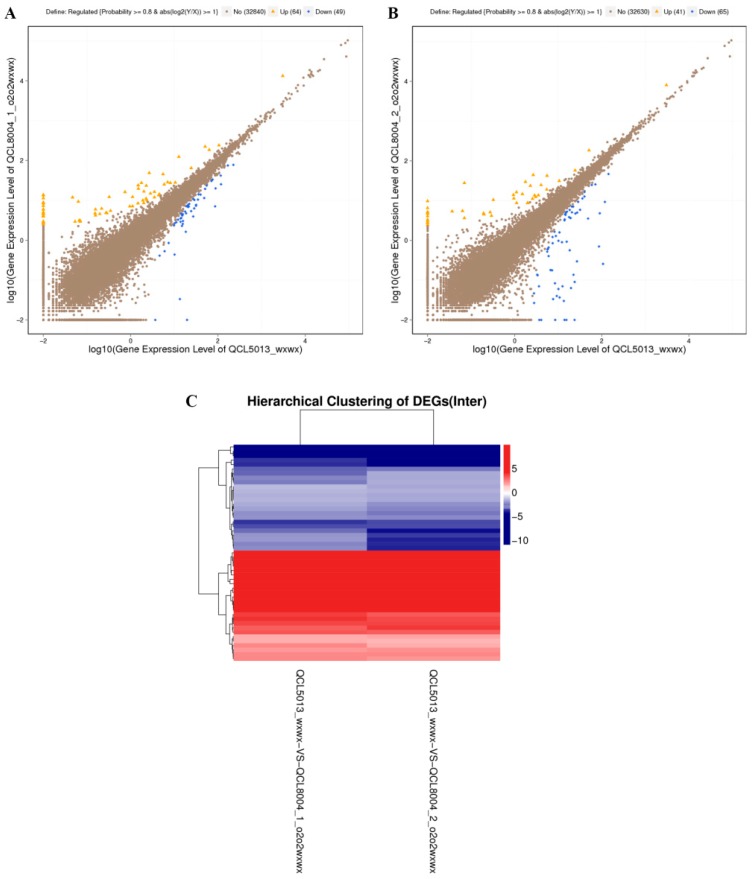
(**A**) Scatter plots of all expressed genes in each pairwise (QCL5013 vs. QCL8004_1). (**B**) Scatter plots of all expressed genes in each pairwise (QCL5013 vs. QCL8004_2). (**C**) Intersection heatmap of DEGs for each cluster plan.

The differentially expressed genes (DEGs) between QCL8004_1, QCL8004_2, and the recurrent parent QCL5013 were analyzed using NOISeq and were hierarchically clustered using Cluster. Compared with their recurrent parent QCL5013, 113 DEGs, including 49 down-regulated and 64 up-regulated genes, were detected in QCL8004_1 (Figure 4A), while 106 DEGs, including 65 down-regulated and 41 up-regulated genes, were detected in QCL8004_2 (Figure 4B). To further reduce the differences caused by different background recovery rates and genetic recombination, the intersection of DEGs (including 25 up-regulated and 24 down-regulated genes) in QCL8004_1 and QCL8004_2 was acquired. This method filtered the most irrelevant DEGs (Figure 4C), allowing for further analysis of actual DEGs.

### 2.5. GO Annotation and KEGG Analysis

**Figure 5:**
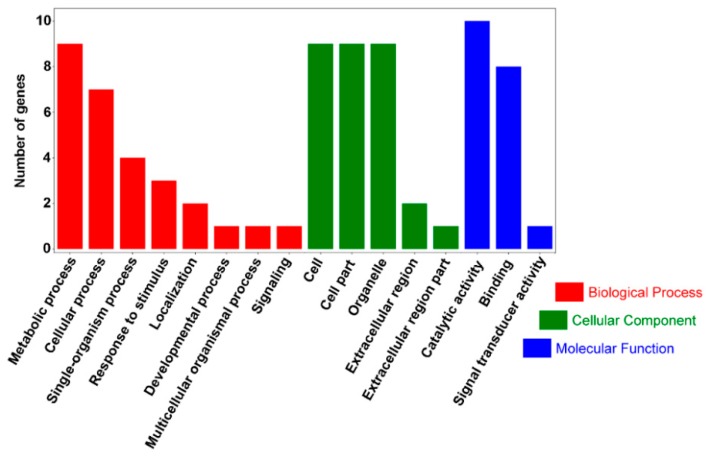
GO functional classification on DEGs for QCL5013 vs. QCL8004_1 and QCL5013 vs. QCL8004_2. Y axis means number of DEGs (the number is presented by its square root value). X axis represents GO terms. All GO terms are grouped into three ontologies: orange is for biological process, brown is for cellular component, and blue is for molecular function.

Gene ontology (GO) functional classification was performed for 49 significantly different genes; the results shown in Figure 5 and Table S7 were related to cell components (CC), molecular function (MF), and biological processes (BP). In CC, 10 genes were detected, which were involved in cells (GO: 0005623), cell parts (GO: 0044464), and organelles (GO: 0043226); in MF, 14 genes detected were involved in binding (GO: 0005488) and catalytic activity (GO: 0003824); in BP, 15 genes detected were involved in cellular processes (GO: 0009987) and metabolic processes (GO: 0008152), including nine genes involved in metabolic processes. Significantly different genes were mainly annotated into MF and BP, indicating that the *o2* gene introgression into waxy corn primarily caused expression changes in genes related to catalytic activity and metabolic processes.

**Figure 6:**
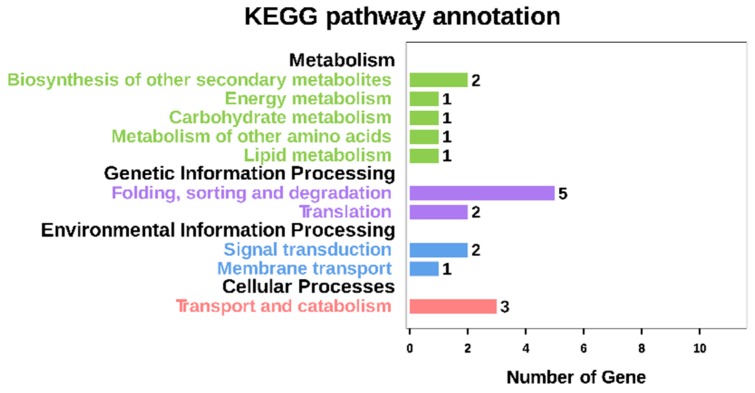
KEGG functional classification of DEGs for QCL5013 vs. QCL8004_1 and QCL5013 vs. QCL8004_2. X axis means number of DEGs. Y axis represents second KEGG pathway terms. All second pathway terms are grouped in top pathway terms indicated in different colors.

Pathway analysis of DEGs based on the Kyoto Encyclopedia of Genes and Genomes (KEGG) database showed that only 18 genes were annotated into 14 known KEGG pathways, while 31 DEGs were not annotated into any known KEGG pathways. As shown in Figure 6 and Table S8, six genes were annotated into metabolism, six genes into genetic information processing, three genes into environmental information processing, and three genes into cellular processes. In taurine and hypotaurine metabolism (KEGG 00430), *Zm00001d033489.1*, which encodes the key enzyme in the conversion of cysteine into hypotaurine (cysteine oxidase), was down-regulated in the endosperm of *o2o2wxwx* double mutants, thereby inhibiting hypotaurine synthesis. In starch and sucrose metabolism (K00500), *Zm00001d033556.1* (K13648), which encodes the key element of pectin synthesis (galacturonosyltransferase), was up-regulated, thereby promoting pectin synthesis. In the phenylpropene (K00940) and flavonoid (K00941) metabolic pathways, *Zm00001d037073.1 (hct3, hydroxycinnamoyl transferase3*) and *Zm00001d045145.1*, which encode the down-stream enzymes of phenylalanine and tyrosine metabolism (hydroxycinnamoyl transferase and 2-oxoglutarate-dependent dioxygenase, respectively), were down-regulated, thereby inhibiting the degradation of phenylalanine and tyrosine.

### 2.6. qRT-PCR Validation

**Figure 7:**
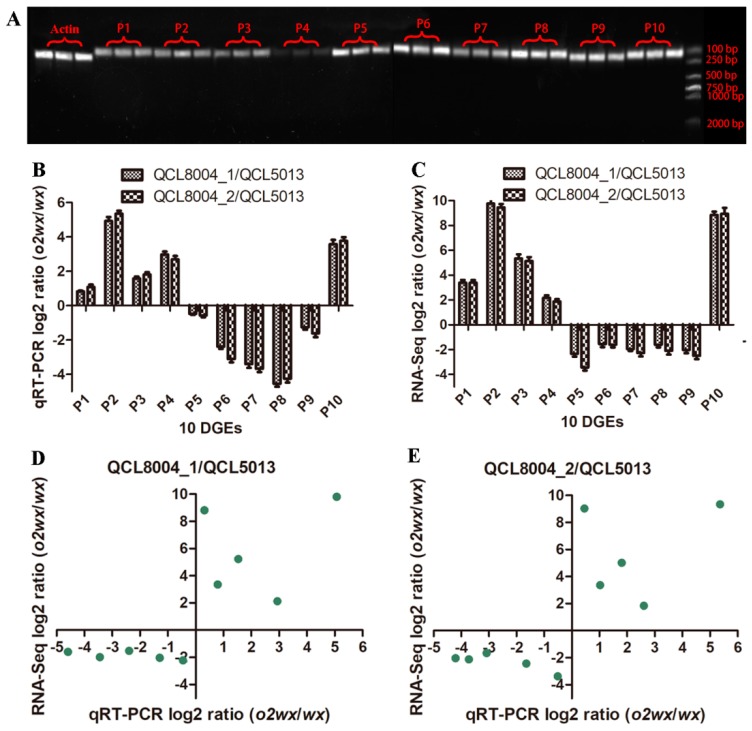
(**A**) The electrophoresis pattern of 10 DGEs by qRT-PCR. (**B**) The qRT-PCR log2 ratio (*o2wx*/*wx*). (**C**) The RNA-Seq log2 ratio (*o2wx*/*wx*) of 10 DGEs. (**D**) The qRT-PCR validation of 10 DGEs identified by RNA-seq, Pearson’s *r* = 0.8760. (**E**) The qRT-PCR validation of 10 DGEs identified by RNA-seq. Pearson’s *r* = 0.8843. P1, *Zm00001d034771.1*; P2, *Zm00001d029385.1*; P3, *Zm00001d012262.1*; P4, *Zm00001d033483.1*; P5, *Zm00001d011063.1*; P6, *Zm00001d028561.1*; P7, *Zm00001d039935.1*; P8, *Zm00001d039936.1*; P9, *Zm00001d028408.1*; P10, *Zm00001d030185.1*.

To validate the expression differences between *o2wx* and *wx* observed by RNA-Seq, we conducted quantitative real-time polymerase chain reaction (qRT-PCR) verification for 10 DGEs. Figure 7A shows that the designed primers for 10 DEGs showed better specificity. Meanwhile, the results shown in Figure 7B demonstrated that the qRT-PCR expression patterns of these 10 genes were similar to those measured by transcriptional sequencing (Figure 7C) between QCL8004_1 vs. QCL5013 and QCL8004_2 vs. QCL5013. In addition, Pearson coefficients of QCL8004_1 vs. QCL5013 and QCL8004_2 vs. QCL5013 were 0.8760 and 0.8843, respectively (Figure 7D,E). The RNA-seq results showed high relevance to the qRT-PCR results, indicating data reliability.

## 3. Discussion

In this study, the *o2* gene was introgressed into waxy corn using the MABS technology to generate *o2* and *wx* double recessive mutants. At the milking stage (18th DAP), the RNA-Seq analysis was conducted on kernels. In the *o2o2wxwx* mutants, the RNA-Seq results revealed 49 DEGs, which included the previously reported elongation factor and heat-shock protein genes [14]. New DEGs were also identified, such as the lysine/histidine specific amino acid transporter gene (*Zm00001d012262.1*, LHT1, lysine histidine transporter 1), a sulfur-rich protein gene (*Zm00001d011063.1*), and a dehydrin gene (*Zm00001d033483.1*, ZmDHN13, dehydrin13). This data will be helpful in revealing the transcriptional regulation mechanisms increasing lysine content after *o2* gene introgression into waxy maize.

In this study, we found that lysine contents of QCL8004_1 and QCL8004_2 were higher than their recurrent parent by 15.63% and 18.75%, respectively; however, these values were lower than those of the mutants acquired by Zhou et al. (0.47%) [14]. Meanwhile, the background recovery rates of QCL8004_1 and QCL8004_2 were 93.16% and 93.28%, respectively, higher than the theoretical recovery rate (87.5%), and slightly higher than the BC_6_F_3_ mutants of Zhou et al. [14]. This was caused by background selection while screening the BC_1_F_1_, BC_2_F_1_, and BC_2_F_2_ generations using the SSR molecular marker technique [14]. In our study, 15,514 polymorphic SNPs were used for background analysis, which was significantly more than the 54 SSR markers used by Zhou et al. These methods favored the elimination of background interference and improved the accuracy of DEGs detection.

In the RNA-seq results, 28307, 28451, 28128, and 29061 genes were detected in QCL5013, QCL8004_1, QCL8004_2, and Tai19, respectively, accounting for over 70% of total corn genes, indicating good coverage of the genome. Differential analysis showed that, compared with the recurrent parent QCL5013, 113 DEGs, including 49 down-regulated and 64 up-regulated genes, were detected in QCL8004_1, while 106 DEGs, including 65 down-regulated and 41 up-regulated genes, were detected in QCL8004_2. Up-regulated and down-regulated genes in QCL8004_1 and QCL8004_2 differed significantly, potentially due to their different genetic background and recombination events. In 2013, Jia et al. [19] reported transcriptional profile differences between the *o2* and wild-type lines. To minimize the influence of such differences, the intersection of DEGs in QCL8004_1 and QCL8004_2 was acquired; this contained 49 significant DEGs, including 25 up-regulated and 24 down-regulated genes. This further improved the accuracy of the DEG analysis.

Based on the GO functional classification statistics, we found that the significant DEGs were mainly involved in MF (14 genes) and BP (15 genes). In addition, pathway analysis based on the KEGG public database showed that 18 genes were annotated to 14 known KEGG pathways. Therefore, the introgression of the *o2* gene into *wxwx* corn resulted in the altered expression of genes associated with MF and BP.

*Zm00001d034771.1*, which encodes elongation factor 2, and *Zm00001d029385.1*, which encodes elongation factor 1-alpha (EF-1α), were up-regulated in the endosperm of *o2o2wxwx* double mutants, agreeing with data from Carneiro et al. [21]. The literature shows that EF-1α levels in corn endosperm highly correlate with lysine content [22]. This could indicate that *Zm00001d029385.1* up-regulation increased EF-1α levels and improved the lysine content. However, it remains to be determined whether the up-regulated expression of *Zm00001d034771.1* was related to the lysine content.

Lactoylglutathione lyase (LGL, also known as glyoxalase I) plays an important role in cellular detoxification and tolerance to abiotic stresses [23]. Li et al. [24] found that two LGLs (GRMZM2G312877 and GRMZM2G028110) were detected as *O2* target genes according to chromatin immune-precipitation followed by sequencing (ChIP-Seq), and thought that *O2* could regulate genes involved in stress resistance. Meanwhile, Zhou et al. [14] studied protein changes in the endosperm of the *o2o2wxwx* double mutant using the two-dimensional gel electrophoresis (2DE) technique. Their results showed that the 17.5 kDa class II heat shock protein (HSP), 17.4 kDa class II heat shock protein, and 17.0 kDa class II heat shock protein were up-regulated in the *o2o2wxwx* endosperm. However, in our study, only *Zm00001d028408.1* (heat shock protein), *Zm00001d039936.1* (16.9 kDa class I heat shock protein 1), *Zm00001d039935.1* (16.9 kDa class I heat shock protein 1), and *Zm00001d028561.1* (class I heat shock protein 3) were detected and found to be down-regulated in the *o2o2wxwx* endosperm. These genes could be regulated by *o2* to influence kernel development.

Literature analysis revealed that amino acids cannot automatically pass through the cell membrane, and so corresponding transport proteins are needed for their transportation [25]. Since the cDNA of the first plant amino acid transporter (NAT2/AAP1) was cloned by Frommer et al. [26] and Hsu et al. [27], several amino acid transporter genes have been identified [28,29,30,31,32]. Chen et al. [31] identified a new lysine/histidine specific amino acid transporter (LHT1) located in roots, young leaves, flowers, and fruit. In this study, *Zm00001d012262.1*, encoding a lysine/histidine-specific amino acid transporter, was significantly up-regulated in the *o2o2wxwx* endosperm, indicating that the *o2* gene introgression into *wxwx* corn could up-regulate the expression of the *LHT1* gene. This was associated with increased lysine transport to the endosperm and grain lysine content. In tryptophan metabolism, the up-regulation of *Zm00001d030185.1* could enhance tryptophan synthesis and increase the grain tryptophan content. Meanwhile, *Zm00001d033483.1*, encoding the ZmDHN13 protein, was up-regulated in the endosperm of the *o2o2wxwx* double mutants. The ZmDHN13 protein contained 15% histidine, 14% glycine, 23.4% lysine, and 14% glutamic acid [31]. The introgression of the *o2* gene into waxy corn promoted the synthesis of the ZmDHN13 protein and increased the lysine content. ZmDHN13 belongs to the dehydrin protein family [33], meaning that increased ZmDHN13 synthesis is also likely to improve the dehydration rate of grains and the plant’s resistance to abiotic stresses, such as low temperature, drought, and high salt. In addition, sulfur-rich proteins are mostly the plant seed prolamin, such as the 10 kDa and 15 kDa zeins of maize [34,35]. In this study, *Zm00001d011063.1*, encoding a sulfur-rich protein, was down-regulated in the endosperm of the *o2o2wxwx* double mutants, indicating that the *o2* gene introgression into *wxwx* corn decreased sulfur-rich protein synthesis and, therefore, increased the lysine content of maize grains.

**Figure 8:**
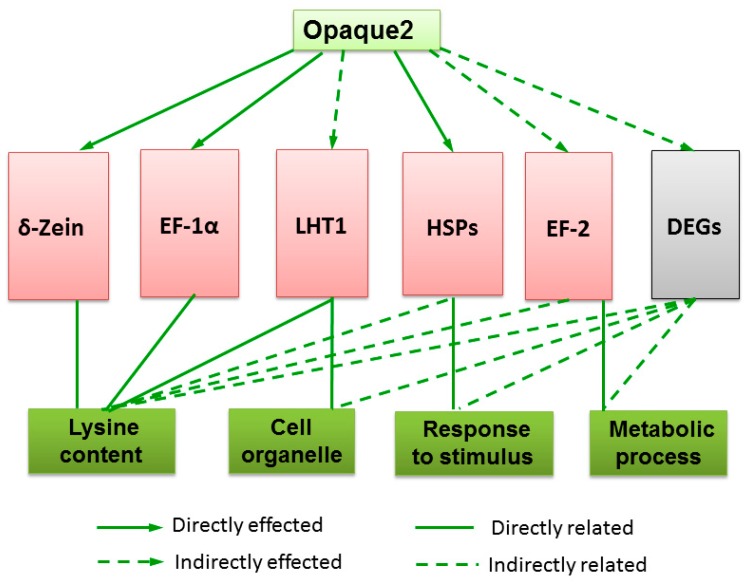
A proposed model of the transcriptional regulatory framework for the *O2* gene in waxy maize. Solid arrows, directly effected; dotted arrows, indirectly effected; full lines, directed related; dotted lines, indirectly related.

Moreover, the *O2* not only directly activates genes associated with a variety of nutrient storage genes and enzymes involved in carbon and amino acid metabolism pathways, but also controls transcription factors that regulate various other aspects of plant metabolism [24]. EF-1α and LHT1 levels in corn endosperm highly correlate with lysine content, and show increased transcription [22,31], while 10 kDa zein shows decreased transcription [24]. Our results demonstrated that the *O2* gene regulated multiple metabolic pathways related to BP and MP during waxy maize endosperm development. In the *o2o2wxwx* kernels, *Zm00001d029385.1* and *Zm00001d012262.1*, which encode the EF-1α and LHT1, respectively, were up-regulated, while *Zm00001d011063.1*, which encodes sulfur-rich proteins (mostly 10 kDa zein), was down-regulated. These changes can improve the grain lysine content of waxy maize, although the functions of these genes in lysine biosynthesis or transport need to be further studied. Meanwhile, the *O2* can directly or indirectly activate HSPs and EF2, and other DEGs associated with response to stimulus, the metabolic process, and other aspects of plant metabolism (Figure 8). These results are important for elucidating the underlying molecular mechanisms in the lysine content improvement of waxy corn following the *o2* gene introgression. The above genes that reduce zeins and increase the lysine content may be used for gene editing or the development of new biomarkers to promote molecular quality breeding.

## 4. Materials and Methods

### 4.1. Materials

QCL5013 (*wxwx*) is a waxy maize inbred line generated by our research group. Tai19 (*o2o2*) is a high lysine maize inbred line from Shanxi Academy of Agricultural Sciences, Shanxi, China. We used QCL5013 (*wxwx*) as the female parent and Tai19 (*o2o2*) as the male parent, to generate an F_1_ hybrid. BC_1_F_1_ was then obtained by backcrossing the F_1_ hybrid to the QCL5013 (recurrent parent). From BC_1_F_1_, 29 plants with the *O2o2wxwx* genotype were identified after the screening of 213 plants using the *wx* gene marker phi027 and *o2* gene marker umc1066 (Table S9); BC_2_F_1_ was then obtained by backcrossing with QCL5013. From BC_2_F_1_, 13 plants with the *O2o2wxwx* genotype were identified after the screening of 50 plants using the *o2* gene marker umc1066; BC_2_F_2_ was then obtained by self-pollination. In total, 110 individual plants from the BC_2_F_2_ generation were planted; plants with the *o2o2wxwx* genotype were screened using umc1066 and BC_2_F_3_ seeds were obtained by self-pollination. These plants continued to be planted, self-pollinated, and purified. Overall, 23 BC_2_F_3_ plants were selected according to their agronomic characteristics. Meanwhile, two BC_2_F_3_ plants, F_3-4_ and F_3-7_, were selected according to the combination of genetic background analysis using 41 SSR markers and a lysine content analysis of 23 plants (Table S10). Self-pollination for additive generations was continued until these lines were genetically stable; plants from these two lines were named QCL8004_1 and QCL8004_2 (Figure S4).

The characteristics and appearance photographs of ears and kernels were taken by a Canon PC1472. Mature kernels were peeled with a razor blade. Then, a small piece of endosperm was spray-coated with the ion sputter (E-1010, Hitachi, Japan). The submicroscopic structure of the endosperm was observed by scanning electron microscopy (S-3400N, Hitachi, Japan) in the Guizhou Key Laboratory of Agricultural Biotechnology Guiyang, China.

### 4.2. DNA Extraction and Genetic Background Analysis

QCL8004_1, QCL8004_2, and their parents (QCL5013 and Tai19) were planted in a test field. Leaves were sampled from five plants per line and genomic DNA was extracted according to Yang’s method [36]. Genotyping these lines was conducted using a 55K SNP chip designed by Cheng et al. [37] on the Affymetrix^®^ GenTian^®^ platform (CapitaBio Technology Co., Ltd. Beijing, China) to measure the genetic background recovery rates of QCL8004-1 and QCL8004-2. Statistical analysis of the background recovery rate based on molecular markers was calculated by the following Formula (1):

(1) $$G\;\left(g\right)=\frac{L+X\;\left(g\right)}{2L}$$

where G (g) is the background recovery rate after backcrossing g generations; X (g) is the number of markers showing band forms of the recipient parent; g represents the generations of backcrossing; and L is the total number of tested markers [38,39,40]. 

The background recovery rate was theoretically calculated using the following Formula (2):

(2) $$E\;\left[G\;\left(g\right)\right]=1-{\left(1/2\right)}^{g+1}$$

### 4.3. RNA-Seq Library Construction and Sequencing

All maize materials were grown in the experimental station (N 26°30′14″ and E 106°39′21″) of the Guizhou Institute of Upland Food Crops, Guizhou Academy of Agricultural Science, China, during the summer of 2016. A minimum of three well-filled ears of each genotype were sampled at the 18th DAP. Total RNA was extracted with the Plant RNA Kit, according to the manufacturer’s instructions (OMEGA); three biological replicates were adopted in this study. The concentration and purity of RNA samples were examined using a NanoDrop 1000 and Agilent 2100 Bioanalyzer. Oligo (dT) magnetic beads were used to select mRNA with a polyA tail, the mRNA was hybridized with a DNA probe, and the DNA/RNA hybrid strand was digested with RNase H, followed by DNase I reaction to remove the DNA probe. The target RNA was fragmented with broken buffer and reversely transcribed to form cDNA using N6 random primers, and the second strand of the cDNA was then synthesized to generate dsDNA. The ends of the dsDNA were repaired, phosphorylated at the 5’ end, and adhered to ‘A’ at the 3’ end; then an adaptor with stretched ‘T’ at the 3’ end was ligated as the PCR substrate. Two specific primers were used to amplify the ligation product. The PCR product was thermally denatured and the single-stranded DNA was cyclized by splint oligo and DNA ligase. Transcriptional sequencing was performed on the BGISEQ-500 platform for the following data analysis.

### 4.4. Identification of Differentially Expressed Genes

Among the raw sequencing reads, the reads with adaptors, the reads in which unknown bases were more than 10%, and the low quality reads (the percentage of low quality bases was over 50% in a read; the low quality base was no more than 5 (Q ≤ 5)) were removed. The remaining reads were clean reads.

For mapping clean reads, Bowtie2 [41] was used to reference genes and HISAT [42] was used to reference the genomes. The FPKM method was used in calculating the expression level by RSEM [43] and Formula (3) was employed, as shown below:

(3) $$\mathrm{FPKM}\left(A\right)=\frac{10^{6}C}{NL/10^{3}}$$

where *C* was the number of fragments that are aligned to gene A; *N* was the total number of fragments that are aligned to all genes; and *L* was the number of bases on gene A. The NOISeq method [44] was used for screening DEGs according to the following default criteria: Fold change ≥ 2 and divergent probability ≥ 0.8.

### 4.5. GO and Pathway Enrichment Analysis of DEGs 

All DEGs were mapped to each term of the gene ontology database (http://www.geneontology.org/) (30 November 2016), and the number of genes in each term was calculated. The hypergeometric test was used to find out which GO items were significantly enriched in the DEGs compared with the whole genome background. Referring to the software GO::TermFinder (http://www.yeastgenome.org/help/analyze/go-term-finder) (30 November 2016), the *p* value was calculated by using Formula (4):

(4) $$P=1-\overset{m-1}{\underset{i=0}{\sum }}\frac{\left(\begin{array}{c} M\\ i \end{array}\right)\left(\begin{array}{c} N-M\\ n-i \end{array}\right)}{\left(\begin{array}{c} N\\ n \end{array}\right)}$$

where *N* was the number of all genes with GO annotation; *n* was the number of DEGs in *N*; *M* was the number of all genes that are annotated to a certain GO term; and m was the number of DEGs in M. After being corrected by Bonferroni correction [45], the calculated *p* value was defined as the GO term. The GO terms with a *p* value ≤ 0.05 were defined as significantly enriched GO terms in DEGs.

KEGG [46] was used to perform the pathway enrichment analysis of DEGs. The formula used to calculate KEGG was similar to that used for GO analysis; here, *N* was the number of all genes with KEGG annotation; *n* was the number of DEGs in *N*; *M* was the number of all genes annotated as specific pathways; and *m* was the number of DEGs in *M*. The pathways with a *q* value ≤ 0.05 were defined as significantly enriched pathways in DEGs.

### 4.6. qRT-PCR Validation

Ten DEGs were selected for qRT-PCR verification. The primers shown in Table 2 were designed online (http://sg.idtdna.com/primerquest/Home/Index), and the first-strand cDNAs were synthesized from 1000 ng of total RNA using a reverse transcription kit (RevertAid First Strand cDNA Synthesis Kit, K1622, Thermo Fisher Scientific Inc., Shanghai, China) in a 20 μL reaction volume. The qRT-PCR was conducted on the CFX Connect Real-Time PCR System (Bio-Rad Laboratories. Inc., Shanghai, China), according to the method used by Liu et al. [47] in a 20 μL reaction volume with the SYBR^®^ Select Master Mix (Applied Biosystems Inc., California, USA), using 4 μL of a tenfold diluted cDNA solution, 10 μL of SYBR^®^ Select Master Mix, and 10 μM of each primer. The thermal cycling conditions were 2 min at 50 °C and 10 min at 95 °C, followed by 40 cycles of 15 s at 95 °C and 1 min at 60 °C. 

The relative expression levels for each gene were calculated using the comparative Ct method with normalization to the internal control gene. Three replicates were used for each sample and actin was used as an internal standard, and the relative expression level was calculated using the 2^−ΔΔC^_T_ method [48].

### 4.7. Accession Numbers

The raw data of RNA-seq reads were deposited in the National Center for Biotechnology Information (NCBI) database under accession number (SRP157066). Biosample accessions were as follows: SAMN09791929, SAMN09791930, SAMN09791931, SAMN09791932, SAMN09791933, SAMN09791934, SAMN09791935, SAMN09791936, SAMN09791937, SAMN09791938, SAMN09791939, and SAMN09791940.

## 5. Conclusions

In this study, the *opaque2* (*o2*) gene was introgressed into the waxy corn using the MABS technology, and two *o2o2wxwx* lines, QCL8004_1 and QCL8004_2, were acquired. The results showed that lysine contents of QCL8004_1 and QCL8004_2 were improved compared to that of their recurrent parent. Meanwhile, NOISeq differential expression analysis showed that 49 differentially expressed genes were mostly related to the catalytic activity and metabolic processes. *Zm00001d029385.1* and *Zm00001d012262.1*, which encode the EF-1α and LHT1, respectively, were up-regulated, while *Zm00001d011063.1*, which encodes sulfur-rich proteins (mostly 10 kDa zein), was down-regulated in the *o2o2wxwx* lines. These changes can improve waxy maize grain lysine content. This information will help uncover the regulatory transcriptional mechanism for the increase of lysine content by *o2* gene introgression into waxy maize.

## Figures and Tables

**Table 1:** The contents of lysine and amylopectin of QCL8004_1, QCL8004_2, Tai 19, and QCL5013.

No.	Name	Genotype	Lysine (%)	±% to QCL5013	Amylopectin (%)	±% to QCL5013
1	Tai19	*o2o2*	0.43	/	/	/
2	QCL5013	*wxwx*	0.32	/	100	/
3	QCL8004_1	*o2o2wxwx*	0.37	15.63	98.95	−1.05
4	QCL8004_2	*o2o2wxwx*	0.38	18.75	98.64	−1.36

**Table 2:** The qPCR primers of ten DEGs.

NO.	Gene ID	Primer Sequences (5′-3′)	Location of Primer	Amplicon Length (bp)
P1	*Zm00001d034771.1*	F: TGGTGGTGGTTGACTGTATTG	Exon 2	95
		R: CACGGTAAGAACTGGCCTAATC		
P2	*Zm00001d029385.1*	F: CCGATGCATCAAACAGAGAGA	Exon 4	120
		R: TCAGGTCGCTTCCTGAATAAC		
P3	*Zm00001d012262.1*	F: AGTAAAGCAGCTCGTGTTCTAA	Exon 2	103
		R: CAATGTGGTAGTGTCGCAAATC		
P4	*Zm00001d033483.1*	F: GGCATCGTGGAGAAGATCAA	Exon 1	101
		R: TCGCCGTGCTTCTTCTTT		
P5	*Zm00001d011063.1*	F: CAACTATGTAGCGGCGTCTAA	Exon 2	138
		R: ACAGACACAGGCGGTAATG		
P6	*Zm00001d028561.1*	F: AGGAGCACGAGGAGAAGAA	Exon 1	108
		R: CCTTGATCTGGTCTGCCTTG		
P7	*Zm00001d039935.1*	F: TCTGGTTGAGCATCCAATCC	Exon 1	102
		R: TCGTAGAAGACACAGGAAACATC		
P8	*Zm00001d039936.1*	F: GAGAACACCAAGGTGGATCAG	Exon 1 and 2	113
		R: CCAGAGATCTCAATAGCCTTCAC		
P9	*Zm00001d028408.1*	F: CAGGAGAACAGGGACAACAG	Exon 2	149
		R: GGCGACATCGGATCAACTAA		
P10	*Zm00001d030185.1*	F: GAGGCTAAAGAGAAGTGCAAGA	Exon 1	117
		R: CTCCTCTCCAACCCTAGATACA		

## Supplementary Materials

Supplementary materials can be found at http://www.mdpi.com/1422-0067/20/3/684/s1. **Table S1**. Genotyping results of Tai19, QCL5013, QCL8004_1, and QCL8004_2 by 55k SNP array. **Table S2**. Summary for RNA sequencing data of Tai19, QCL5013, QCL8004_1, and QCL8004_2. **Table S3**. Alignment statistic of reads aligned with reference gene. **Table S4**. Alignment statistic of reads aligned with reference genome. **Table S5**. QC20 and QC30 of RNA sequencing data for Tai19, QCL5013, QCL8004_1, and QCL8004_2. **Table S6**. FPKM of all genes for Tai19, QCL5013, QCL8004_1, and QCL8004_2. **Table S7**. GO functional classification on DEGs for QCL5013 vs. QCL8004_1 and QCL5013 vs. QCL8004_2. **Table S8**. KEGG functional classification on DEGs for QCL5013 vs. QCL8004_1 and QCL5013 vs. QCL8004_2. **Table S9.** The information of SSR primers for the *o2* and *wx* alleles. **Table S10**. Background recovery rates and lysine contents of 23 BC_2_F_3_ plants. **Figure S1**. Graphical genotypes of QCL8004_1 and QCL8004_2 on chromosomes 1, 2, 3, 4, and 5. QCL5013, recurrent parent; Tai19, donor parent; A, B, H, and U of the SNP markers were used to establish the database. A, consistent with the recurrent parent; B, consistent with the donor parent; H, heterozygous; U, unidentified. **Figure S2**. Graphical genotypes of QCL8004_1 and QCL8004_2 on chromosomes 6, 7, 8, 9, and 10. QCL5013, recurrent parent; Tai19, donor parent; A, B, H, and U of the SNP markers were used to establish the database. A, consistent with the recurrent parent; B, consistent with the donor parent; H, heterozygous; U, unidentified. **Figure S3**. The heatmap of correlation coefficient values across samples. a, b, c represents three biological replicates. Gradient color barcode at the right top indicates the minimum value in white and the maximum in blue. If one sample is highly similar to another one, the correlation value between them is very close to 1. **Figure S4**. The MABS scheme adapted for the introgression of *o2* gene into the waxy maize inbred QCL5013. MAS, marker-assisted selection; FS, foreground selection; BS, background selection.

## Abbreviations

GBSS-I	Granule-bound starch synthase I
MABS	Marker-assisted backcrossing selection
DAP	Day after pollination
SNP	Single nucleotide polymorphism
SSR	Simple Sequence Repeats
qRT-PCR	Quantitative real-time polymerase chain reaction
FPKM	Fragments Per Kilobase Million
GO	Gene Ontology
KEGG	Kyoto Encyclopedia of Genes and Genomes
hct3	Hydroxycinnamoyl transferase 3
LHT1	Lysine histidine transporter 1
DHN13	Dehydrin 13
EF-1α	Elongation factor 1-alpha
MF	Molecular function
BP	Biological processes
CC	Cell components
ChIP-Seq	Chromatin immune-precipitation follow by sequencing
HSP	heat shock protein
SEM	Scanning electron microscopy
