# Supplementary material for: Molecular Mechanisms Underlying Increase in Lysine Content of Waxy Maize through the Introgression of the opaque2 Allele
Source: Int J Mol Sci. 2019 Feb 5;20(3):684. doi: 10.3390/ijms20030684 (PMC6386912; doi:10.3390/ijms20030684)
Supplement: Supplementary file 1 [file ijms-20-00684-s001.zip › Table S1-S10/Table S10. Background recovery rates and lysine contents of 23 BC2F3 plants..docx]

**Table S10.** Background recovery rates and lysine contents of 23 BC2F3 plants.

| NO. | F_1-14_ | F_1-16_ | F_1-17_ | F_1-19_ | F_1-2_ | F_1-20_ | F_1-22_ | F_1-25_ | F_1-3_ | F_1-8_ | F_2-2_ | F_2-4_ | F_2-6_ |
| --- | --- | --- | --- | --- | --- | --- | --- | --- | --- | --- | --- | --- | --- |
| G (g) | 0.92 | 0.951 | 0.93 | 0.935 | 0.939 | 0.94 | 0.91 | 0.939 | 0.941 | 0.92 | 0.927 | 0.87 | 0.88 |
| Lys (%) | 0.29 | 0.301 | 0.3 | 0.284 | 0.307 | 0.281 | 0.32 | 0.309 | 0.29 | 0.31 | 0.206 | 0.28 | 0.33 |
| NO. | F2-7 | F2-8 | F2-9 | F3-10 | F3-14 | F3-17 | F3-4 | F3-7 | F6-23 | F6-3 | tai19 | 5013 |  |
| G (g) | 0.94 | 0.902 | 0.97 | 0.924 | 0.903 | 0.938 | 0.96 | 0.951 | 0.915 | 0.9 |  |  |  |
| Lys (%) | 0.31 | 0.304 | 0.31 | 0.28 | 0.292 | 0.295 | 0.39 | 0.333 | 0.314 | 0.3 | 0.415 | 0.23 |  |

G (g), the background recovery rate after backcrossing of g generations, G (g) = [L+x(g)]/2L; X (g), number of markers showing band forms of the recipient parent; g, generations of backcrossing; L, total number of tested markers.
