# Supplementary material for: Molecular Mechanisms Underlying Increase in Lysine Content of Waxy Maize through the Introgression of the opaque2 Allele
Source: Int J Mol Sci. 2019 Feb 5;20(3):684. doi: 10.3390/ijms20030684 (PMC6386912; doi:10.3390/ijms20030684)
Supplement: Supplementary file 1 [file ijms-20-00684-s001.zip › Table S1-S10/Table S9. The informaiton of SSR primers for the o2 and wx alleles.docx]

**Table S9**. The informaiton of SSR primers for the *o2* and *wx* alleles

| NO. | Gene | SSR locus | Bin | Primer sequences (5’- 3’) |
| --- | --- | --- | --- | --- |
| 1 | *o2* | umc1066 | 7.01 | F: ATGGAGCACGTCATCTCAATGG  R: AGCAGCAGCAACGTCTATGACACT |
| 2 | *wx* | phi027 | 9.03 | F: CACAGCACGTTGCGGATTTCTCT  R: GCGTACGTACGACGAAGACAC |
