# Supplementary figures and images for: Molecular Mechanisms Underlying Increase in Lysine Content of Waxy Maize through the Introgression of the opaque2 Allele
Source: Int J Mol Sci. 2019 Feb 5;20(3):684. doi: 10.3390/ijms20030684 (PMC6386912; doi:10.3390/ijms20030684)

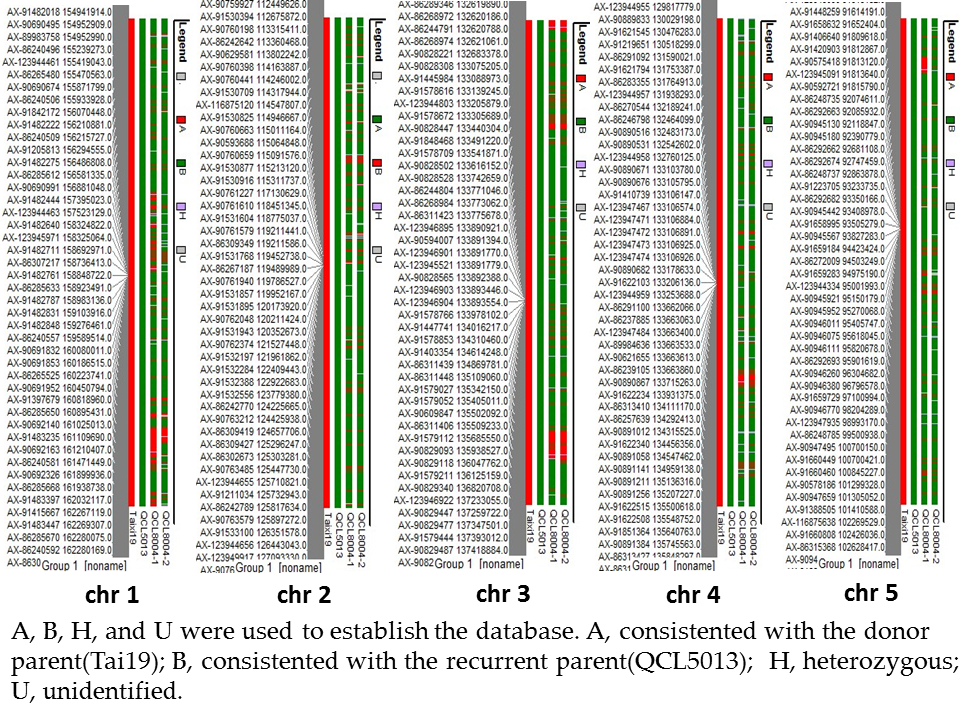

Supplement: Supplementary file 1 [file ijms-20-00684-s001.zip › Figure S1-S4/Figure S1.tif]

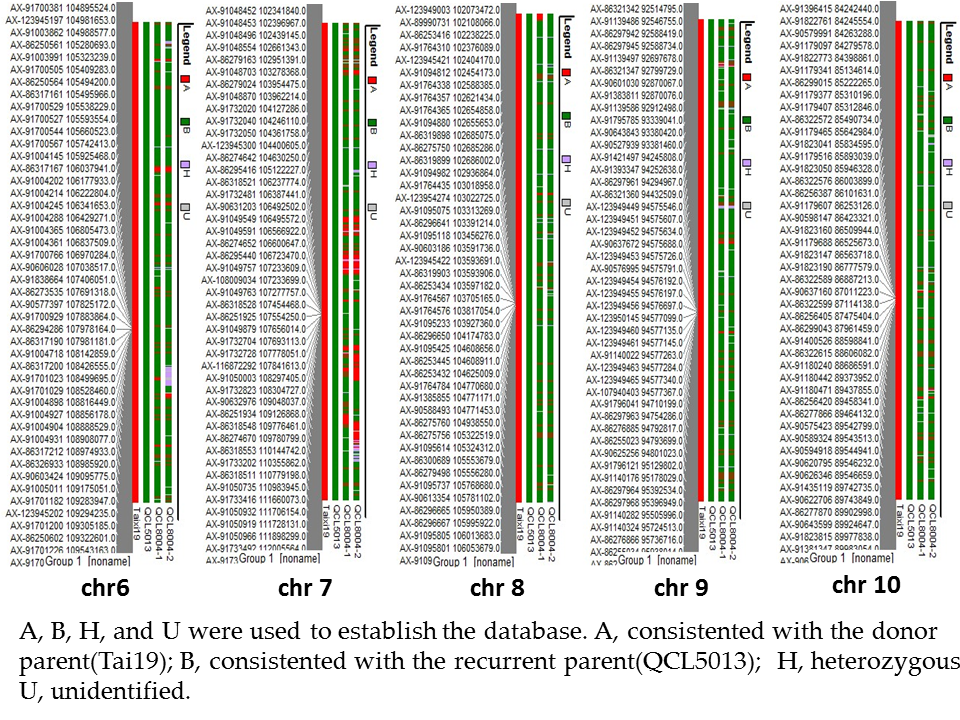

Supplement: Supplementary file 1 [file ijms-20-00684-s001.zip › Figure S1-S4/Figure S2.tif]

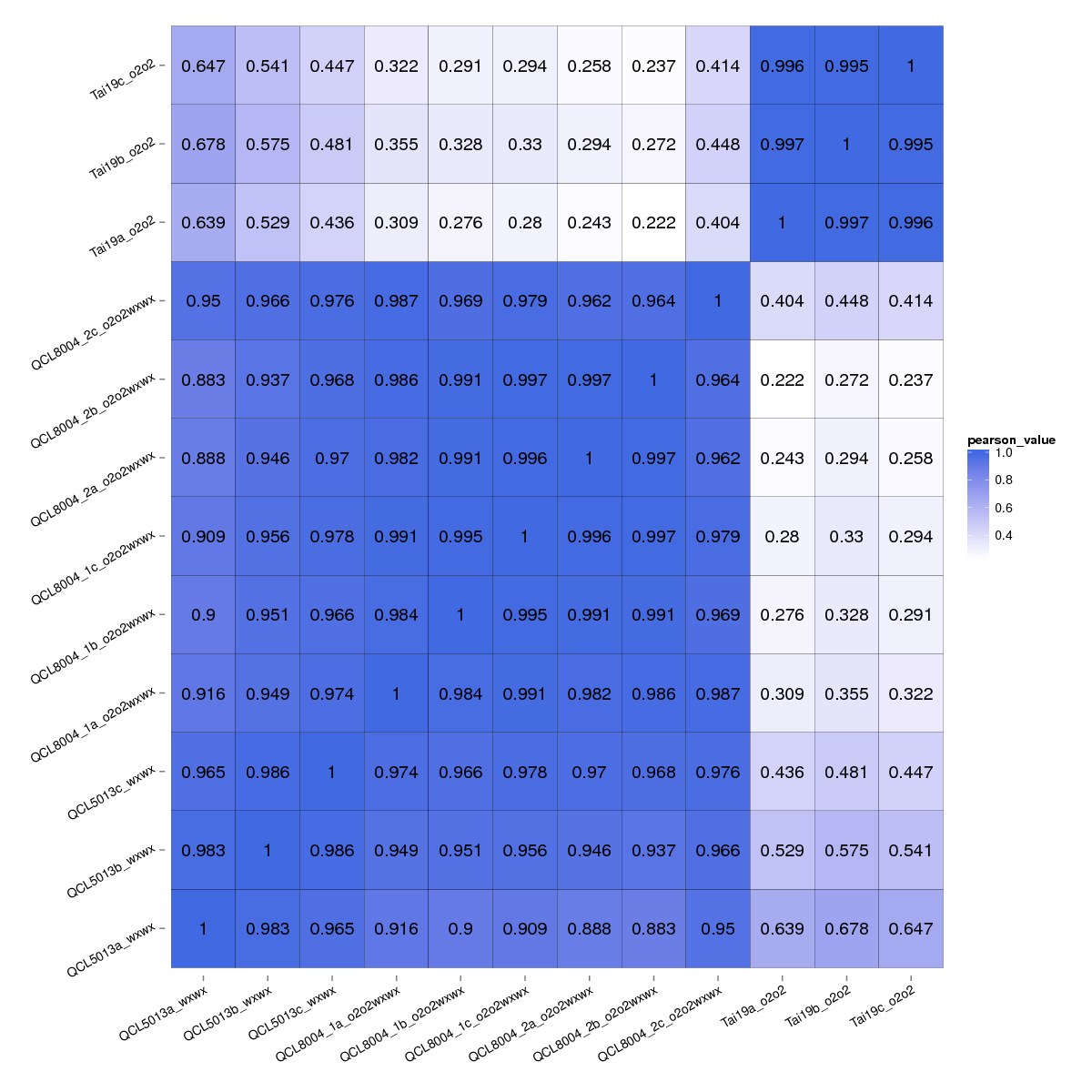

Supplement: Supplementary file 1 [file ijms-20-00684-s001.zip › Figure S1-S4/Figure S3.png]

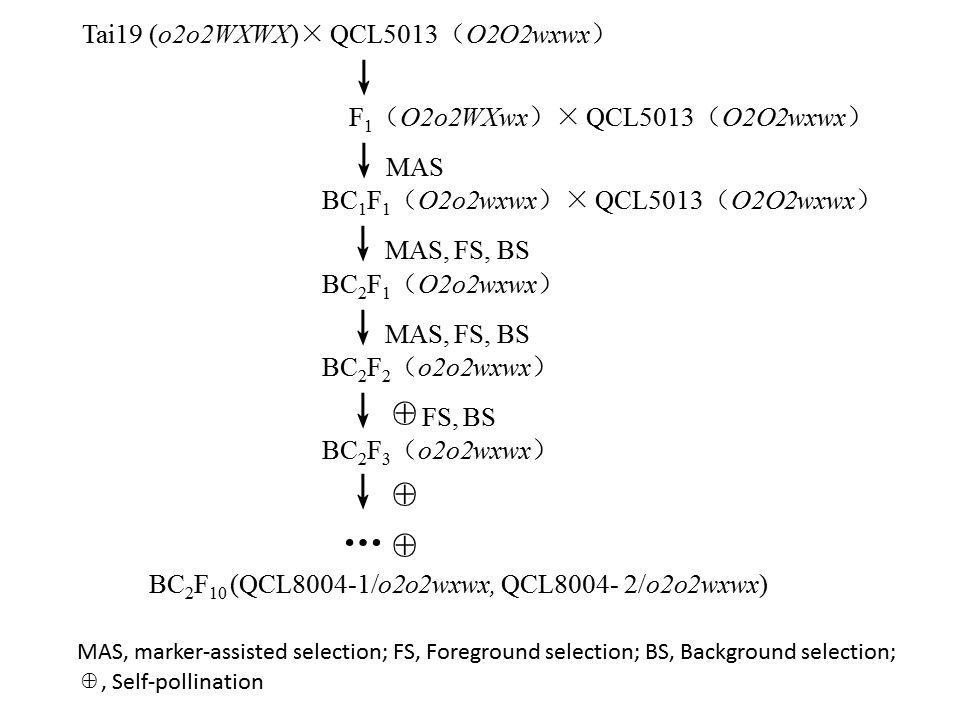

Supplement: Supplementary file 1 [file ijms-20-00684-s001.zip › Figure S1-S4/Figure S4.tif]
